# MRI Plaque Imaging Detects Carotid Plaques with a High Risk for Future Cerebrovascular Events in Asymptomatic Patients

**DOI:** 10.1371/journal.pone.0067927

**Published:** 2013-07-24

**Authors:** Lorena Esposito-Bauer, Tobias Saam, Iman Ghodrati, Jaroslav Pelisek, Peter Heider, Matthias Bauer, Petra Wolf, Angelina Bockelbrink, Regina Feurer, Dominik Sepp, Claudia Winkler, Peter Zepper, Tobias Boeckh-Behrens, Matthias Riemenschneider, Bernhard Hemmer, Holger Poppert

**Affiliations:** 1 Department of Neurology, Technische Universität München, Munich, Germany; 2 Institute for Clinical Radiology, Ludwig-Maximilians-University Hospital Munich, Munich, Germany; 3 Department of Vascular Surgery, Technische Universität München, Munich, Germany; 4 Department of Vascular Surgery, Isar Medizin Zentrum München, Munich, Germany; 5 Department of Neurology, Bethesda Spital Basel, Basel, Switzerland; 6 Institute for Medical Statistics and Epidemiology, Technische Universität München, Munich, Germany; 7 Institute for Social Medicine, Epidemiology, and Health Economics, Charité – University Medicine Berlin, Berlin, Germany; 8 Department of Neuroradiology, Technische Universität München, Munich, Germany; 9 Department of Psychiatry and Psychotherapy, Universitätsklinikum des Saarlandes, Homburg, Germany; University of Cambridge, United Kingdom

## Abstract

**Purpose:**

The aim of this study was to investigate prospectively whether MRI plaque imaging can identify patients with asymptomatic carotid artery stenosis who have an increased risk for future cerebral events. MRI plaque imaging allows categorization of carotid stenosis into different lesion types (I–VIII). Within these lesion types, lesion types IV–V and VI are regarded as rupture-prone plaques, whereas the other lesion types represent stable ones.

**Methods:**

Eighty-three consecutive patients (45 male (54.2%); age 54–88 years (mean 73.2 years)) presenting with an asymptomatic carotid stenosis of 50–99% according to ECST-criteria were recruited. Patients were imaged with a 1.5-T scanner. T1-, T2-, time-of-flight-, and proton-density weighted studies were performed. The carotid plaques were classified as lesion type I–VIII. Clinical endpoints were ischemic stroke, TIA or amaurosis fugax. Survival analysis and log rank test were used to ascertain statistical significance.

**Results:**

Six out of 83 patients (7.2%) were excluded: 4 patients had insufficient MR image quality; 1 patient was lost-to-follow-up; 1 patient died shortly after the baseline MRI plaque imaging. The following results were obtained by analyzing the remaining 77 patients. The mean time of follow-up was 41.1 months.

During follow-up, n = 9 (11.7%) ipsilateral ischemic cerebrovascular events occurred. Only patients presenting with the high-risk lesion types IV–V and VI developed an ipsilateral cerebrovascular event versus none of the patients presenting with the stable lesion types III, VII, and VIII (n = 9 (11.7%) vs. n = 0 (0%) during follow-up). Event-free survival was higher among patients with the MRI-defined stable lesion types (III, VII, and VIII) than in patients with the high-risk lesion types (IV–V and VI) (log rank test *P*<0.0001).

**Conclusions:**

MRI plaque imaging has the potential to identify patients with asymptomatic carotid stenosis who are particularly at risk of developing future cerebral ischemia. MRI could improve selection criteria for invasive therapy in the future.

## Introduction

Carotid artery stenosis (CS) represents a risk factor for cerebral infarction. Pooled data from the Asymptomatic Carotid Atherosclerosis Study (ACAS) [1] and the Asymptomatic Carotid Surgery trial (ACST) [2] revealed that around 89% of the patients did not have a cerebrovascular event for 5 years when treated medically. These data highlight the importance of other criteria than the degree of stenosis, which still represents the main parameter for risk estimation in CS. Histological studies regarding plaque morphology identified specific parameters indicating plaque rupture and have led to the concept of the “vulnerable plaque”: carotid plaques characterized by a thinned fibrous cap with a lipid-rich necrotic core [3], [4], [5] or by intraplaque hemorrhage [6], [7], [8] represent unstable, rupture-prone lesions with a high risk of spontaneous thromboembolic events. Apart from histological studies, high-resolution magnetic resonance imaging (MRI) represents a new noninvasive imaging technique that can reliably identify these key plaque features in vivo [9], [10], [11], [12], [13], [14], [15], [16], [17]. The ability of MRI to visualize such plaque components allows classification of carotid plaques into distinct lesion types (I–VIII) in accordance with the histological American Heart Association (AHA) criteria [18], [19]. Cai and colleagues modified this histological classification specifically for multicontrast MRI application [20]. Using this modified classification, plaques containing a thinned fibrous cap with a lipid-rich necrotic core[21] can be categorized as lesion type IV–V and plaque features such as intraplaque hemorrhage belong to lesion type VI [9], [21].

The ability of MRI plaque imaging to predict cerebral ischemia in asymptomatic patients with CS could improve selection of individuals for invasive therapy. Retrospective studies have already shown a relation between MRI-detected unstable plaque lesions and recent neurological symptoms [22]. In a previous study we found MRI-defined unstable carotid plaques to be clearly overrepresented in patients with symptomatic carotid artery stenosis [23] and especially in diabetic patients [24].

However, prospective studies analyzing the value of MRI plaque imaging for future cerebral infarcts are limited [25].

The aim of this study was to investigate prospectively whether MRI plaque imaging can identify patients with high-risk, asymptomatic CS who have an increased risk for future cerebral events.

## Methods

### Ethics Statement

Written informed consent was obtained from each patient prior to participation. The study protocol was approved by the local ethics committee (Ethikkommission der Fakultät für Medizin der Technischen Universität München). Independent data safety monitoring was provided by the local clinical study center (Münchner Studienzentrum) at the Technische Universität München. Patient data were partially acquired from a pooled data set of patients undergoing MRI plaque imaging [23], [24], [26]. The methods used in the study were in accordance with the ethical standards laid down in the 1964 Declaration of Helsinki.

### Study population

A total of 83 study subjects were recruited for the study from consecutive patients presenting to our neurology department or attending our outpatient clinic. The study started on September 2005 and was completed in July 2010.

Inclusion criteria were (1) internal carotid artery (ICA) stenosis ≥50%, diagnosed by duplex sonography using European Carotid Surgery Trialists' (ECST) criteria [27]; (2) asymptomatic status with regard to their carotid artery disease in their previous medical history. Exclusion criteria were: (1) previous or planned carotid endarterectomy or carotid artery stenting on the index side; (2) previous neck irradiation; (3) contraindications for MRI (e.g., pacemakers, metal implants, claustrophobia).

### Clinical variables

Before undergoing the MRI plaque imaging, each patient was clinically examined and a detailed standardized health questionnaire was completed by a neurologist. The examination included physical status, blood tests, blood pressure measurement and a12-lead electrocardiogram (ECG). Additionally, following diagnostic protocol was used for each patient: We performed a Doppler sonography of the extra- and intracranial vessels as well as a color-coded duplex ultrasound of the extracranial arteries to determine the degree of vessel stenosis. MR- or computed tomography (CT)-angiography was obtained for additional assessment of the degree of stenosis.

Regarding clinical variables, diabetes mellitus (DM) type 2 (DM 2) was defined as a fasting glucose level >7.0 mmol/l (126 mg/dl), glucose level at any time >11.1 mmol/l (200 mg/dl), use of hypoglycemic agents, or a history of physician-diagnosed DM. Hypertension was defined as systolic blood pressure >140 mmHg or diastolic blood pressure >90 mmHg in the supine position, or use of antihypertensive medication because of previously diagnosed hypertension. Hyperlipidemia was defined as a fasting cholesterol value >6.2 mmol/l (240 mg/dl), low-density lipoprotein (LDL) cholesterol >4.9 mmol/l (190 mg/dl), LDL/high-density lipoprotein (HDL) ratio >4.0, or a history of physician-diagnosed increased cholesterol and the use of lipid-lowering medication. Ischemic heart disease was defined as a history of myocardial infarction, angina pectoris, or coronary artery bypass or stenting or a pathognomonic ECG.

The clinical endpoint for the study was a cerebrovascular event including ischemic stroke, transient ischemic attack (TIA), or amaurosis fugax in the region supplied by the index carotid artery.

The clinical endpoints were ascertained every 6–12 months either by physical examination by a neurologist when the study patients attended our outpatient clinic or by telephone interviews using a standardized, detailed health questionnaire. In the case of a suspected cerebral event, either neuroimaging was performed or externally provided hospital records were analyzed. Stroke was considered to be of ischemic origin when cerebral hemorrhage was excluded by MRI or CT. TIA was defined as a new-onset focal neurological abnormality lasting <24 h. Amaurosis fugax was defined as acute onset of transient partial or complete monocular loss of vision.

All patients presenting with carotid artery stenosis at our clinic received as medical treatment 100 mg of aspirin or 75 mg of clopidogrel per day and additionally a statin therapy. In a multidisciplinary conference of neurologists, vascular surgeons and neuroradiologists a consensus decision was reached concerning medical or interventional therapy (carotid endarterectomy or carotid artery stenting) for the patient. The physicians involved were unaware of the MRI plaque imaging findings. Participation in our study did not have any influence on the decision for or against invasive therapy.

### MRI plaque imaging

Each patient was imaged with a 1.5-T scanner (Magnetom Symphony Quantum Gradient; Siemens Medical System, Germany) with bilateral phased-array surface coils (PACC-SS15; Machnet B.V., the Netherlands). According to our previously published protocol, four contrast-weighted images were obtained as follows [23], [24], [26]: three-dimensional time-of-flight MR-angiography (3D TOF), T1-weighted (T1w), T2-weighted (T2w), and proton-density-weighted (PDw) studies of both carotid arteries. The MRI scan was centered on the carotid bifurcation on the side of the stenosis to assure proper matching between the contrast-weighted imaging series of each patient. The imaging sequences were as follows: 3D TOF: field of view (FOV) 200 mm/75.0%; repetition time (TR) 43 ms; time to echo (TE) 7.15 ms, number of excitations (NEX) 2. T1w: FOV 160 mm/100%; TR 700 ms; TE 14 ms; NEX 2. T2w: FOV 160 mm/100%; TR 700 ms; TE 100 ms; NEX 2. PDW: FOV 160 mm/100%; TR 700 ms; TE 10 ms; NEX 2. Slice thickness was 1 mm for the 3D TOF and 2 mm for the T1w, T2w, and PDW images. The longitudinal coverage of each carotid artery was 72 mm (72 slices) for the 3D TOF and 24 mm (12 slices) for T1w, T2w, and PDW images.

The patients were positioned on a vacuum pillow to avoid head–neck region movement during the MRI scan to ensure proper alignment between the images acquired in the four contrast-weighted imaging sequences of each patient.

Before evaluation of the MRI scans, an image-quality rating (4-point scale, 1 = best; 4 = worst) was assigned to all MR images for each contrast-weighted image. Image quality of 4 in one of the contrast weightings led to exclusion of the evaluation procedure. For each patient, a dataset of 108 contrast-weighted MR images of the carotid arteries was obtained (72 slices for the 3D TOF and 12 slices for T1w, T2w, and PDw). The images were evaluated by two reviewers. A consensus decision was reached for each plaque feature.

The reviewers were blinded to the patient's clinical history at the time of image analysis. To determine the lesion type in accordance with the modified AHA criteria [20], the carotid atherosclerotic plaque in the 108 images of each patient was identified and ascribed to one of the six classification types according to the following modified AHA criteria [20]: Type I–II shows near-normal wall thickness without calcification. Type III represents diffuse intimal thickening or small eccentric plaque without calcification. Type IV–V is characterized by a lipid-rich necrotic core surrounded by fibrous tissue with possible calcification. Type VI shows a complex plaque with possible surface defect, hemorrhage, or thrombus. Type VII represents a calcified lesion. Type VIII is characterized by a fibrotic plaque without a lipid core and with possible small calcifications.

### Statistical analysis

Event rates were investigated using survival analysis and log rank test to compare the occurrence of new neurological events and MRI-defined lesion types. The Kaplan–Meier product limit method was used to estimate cumulative event-free rates for graphical display depending on the presence of MRI-defined high-risk lesion types. Log rank test was used to compare the survival distribution between patients with stable lesion types vs. patients with unstable lesion types. Fisher's Exact test and independent sample t-test were performed to determine differences between the group of patients presenting with stable lesion types and the group of patients presenting with unstable lesion types regarding cerebrovascular risk factors and age. The prognostic value of the different plaque components was calculated using survival analysis and log rank test to compare the occurrence of new neurological events and MRI-defined plaque components/lesion types.

Data were analyzed using SPSS version 21.0 software (SPSS, Chicago, IL, USA). All tests were two-tailed and *P*-values<0.05 were considered statistically significant.

## Results

Among the 83 patients (45 male (54.2%); age 54–88 years (mean 73.2 years)) available, 4 were excluded because of insufficient MR image quality; 1 patient was lost-to-follow-up; 1 patient died shortly after the baseline MRI plaque imaging because of renal failure (this patient did not have a neurological event during follow-up). The following results were obtained by analyzing the remaining 77 patients. Forty-eight (62.3%) of these 77 patients were seen in our outpatient clinic for follow-up; 29 (37.7%) patients were followed up by telephone interview.

The mean time of follow-up was 41.1 months (median 42 months, range 12–58 months). During this period, 9 (11.7%) of the 77 patients developed an ipsilateral ischemic cerebrovascular event (1 TIA, 8 ischemic strokes).

Thirteen patients (16.9%) patients presented with bilateral stenosis. In cases of bilateral stenosis, we evaluated the carotid plaque with the more advanced stenosis, so in total 77 carotid plaques were evaluated. Eleven patients presented with severe stenosis on one side and moderate stenosis on the other side; two patients presented with bilateral severe stenosis. Patient characteristics and baseline data are summarized in Table 1.

**Table 1:** Baseline demographic characteristics of study population (77 patients).

Variable	
Age, years (mean)	54–88 (72.8)
Sex, male	n = 42 (54.5%)
Hypertension	n = 60 (77.9%)
Atrial fibrillation	n = 2 (2.6%)
Current or former smoker	n = 38 (49.4%)
Hypercholesterolemia	n = 49 (63.6%)
Diabetes mellitus Type 2	n = 25 (32.5%)
Coronary heart disease	n = 23 (29.9%)
Degree of stenosis <70% (ECST)	n = 24 (31.2%)
Degree of stenosis >70% (ECST)	n = 53 (68.8%)

### MRI lesion types

Patient demographic variables in the group of patients with MRI-defined stable and unstable lesion types are summarized in Table 2.

**Table 2:** Demographic variables in the group of patients with MRI-defined stable and unstable lesion types.

Variable	Patients with MRI-defined	Patients with MRI-defined	*P*-value
	Stable Lesion Types	Unstable Lesion Types	
n (%)	41 (53.2%)	36 (46.8%)	
Age, years (mean)	54–87 (72.3)	55–88 (73.4)	NS (0.73)
Sex, male	22(53.7%)	20 (55.6%)	NS (0.35)
Hypertension	32 (78.0%)	28 (77.8%)	NS (0.79)
Atrial fibrillation	1 (2.4%)	1 (2.8%)	NS (1.0)
Current or former smoker	22 (53.7%)	16 (44.4%)	NS (0.50)
Hypercholesterolemia	28(68.3%)	21 (58.3%)	NS (0.48)
Diabetes mellitus Type II	10 (24.4%)	15 (41.7%)	NS (0.14)
Coronary heart disease	12 (29.3%)	11 (30.6%)	NS (1.0)
Degree of stenosis <70% (ECST)	9 (22.0%)	15 (41.7%)	NS (0.09)
Degree of stenosis >70% (ECST)	32 (78.0%)	21 (58.3%)	NS (0.09)

NS: Not significant.

**Figure 1:**
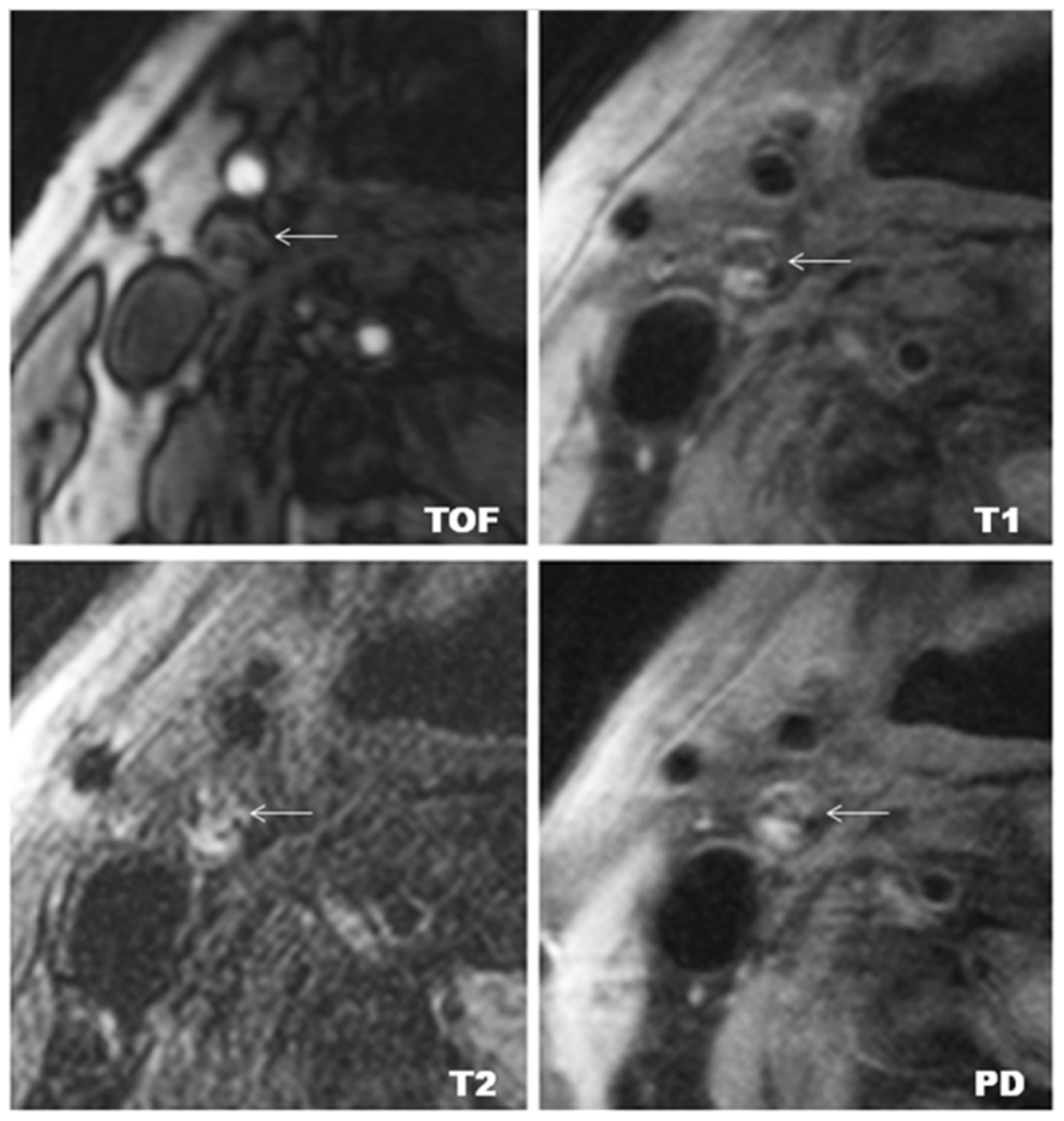
Example of lesion type IV–V in the right internal carotid artery. Lesion type IV–V is characterized by a lipid-rich necrotic core; (←) indicates carotid plaque. The lipid-rich necrotic core shows low- to iso-signal intensity on TOF, T1w, PDw, and T2w images. Original magnification ×25.

**Figure 2:**
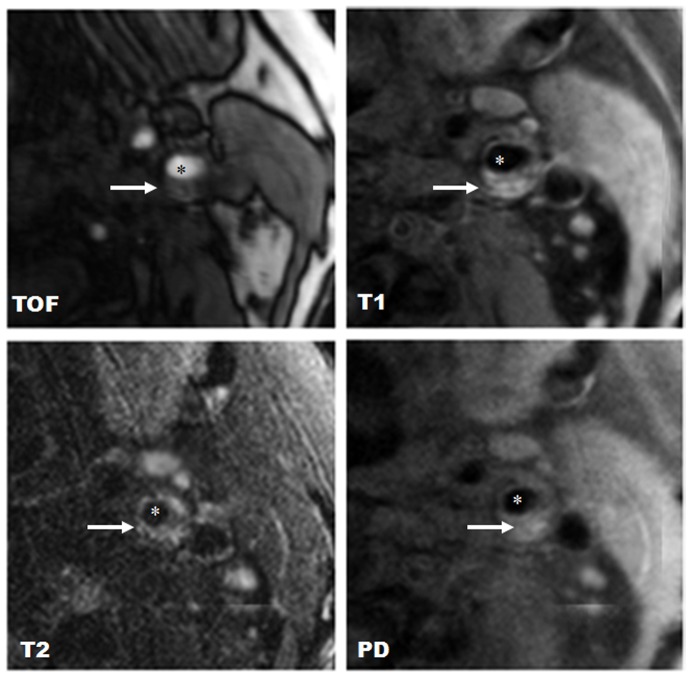
Example of lesion type VI in the left internal carotid artery. Lesion type VI is characterized by intraplaque hemorrhage; (*) indicates the lumen of the carotid artery; (←) indicates carotid plaque. Intraplaque hemorrhage shows high signal intensity on T1w, TOF, PDW, and T2w images. Original magnification ×25.

Lesion type III was found in two carotid plaques (2.6%); lesion type IV–V was found in 16 carotid plaques (20.8%); lesion type VI was found in 21 carotid plaques (27.3%); lesion type VII was found in 35 carotid plaques (45.5%); and lesion type VIII was found in 3 carotid plaques (3.9%). Figure 1 shows a representative case of lesion type IV–V. Figure 2 shows a representative case of lesion type VI.

Only patients presenting with the high-risk lesion types IV–V and VI developed an ipsilateral cerebrovascular event versus none of the patients presenting with the stable lesion types III, VII, and VIII (n = 9 (11.7%) vs. n = 0 (0%) during follow-up). The carotid plaques of 4 (44.4%) of these 9 patients who developed an ischemic event during follow-up were classified as lesion type IV–V, 5 (55.6%) of these patients presented with lesion type VI.

Only plaques containing distinct features we categorized as unstable (lipid-rich necrotic core; thinned/ruptured fibrous cap; intraplaque hemorrhage) showed a significant association with future ischemic cerebral events: Plaques showing intraplaque hemorrhage were associated with the development of cerebral ischemia (n = 5 during follow-up; log rank test *P*<0.001) and plaques containing a lipid-rich-necrotic core or a thinned/ruptured fibrous cap were also associated with the development of cerebral ischemia (n = 4 during follow-up; log rank test *P*<0.05); the other plaque components (diffuse intimal thickening, extensive calcification, fibrotic plaque) were not associated with new cerebral events (n = 9 (11.7%) vs. n = 0 (0%) during follow-up).

Event-free survival was higher among patients with the MRI-defined stable lesion types (III, VII, and VIII) than in patients with the MRI-defined high-risk lesion types (IV–V and VI) (58 months event-free probability 100% vs. 67.8%; log rank test P<0.0001). On analysis of the relation between ischemic stroke alone (without TIA) and MRI-detected high-risk lesion types, the presence of the high-risk lesion types IV–V and VI was still related to ipsilateral cerebrovascular events (n = 8 (10.4%) vs. n = 0 (0%) during follow-up; log rank test *P*<0.001).

Two (22.2%) of the nine patients who developed a cerebral event during follow-up presented with a moderate stenosis (<70% using ECST criteria), whereas 7 (77.8%) of the patients developing cerebral ischemia showed an advanced carotid stenosis (>70% according to ECST criteria). Controlling for cerebrovascular risk factors (DM 2, cholesterol level, hypertension, atrial fibrillation, smoking status, and coronary heart disease) using multivariable analyses (Cox regression) was not possible in our study due to the low number of events (n = 9 (11.7%)) [28].

**Figure 3:**
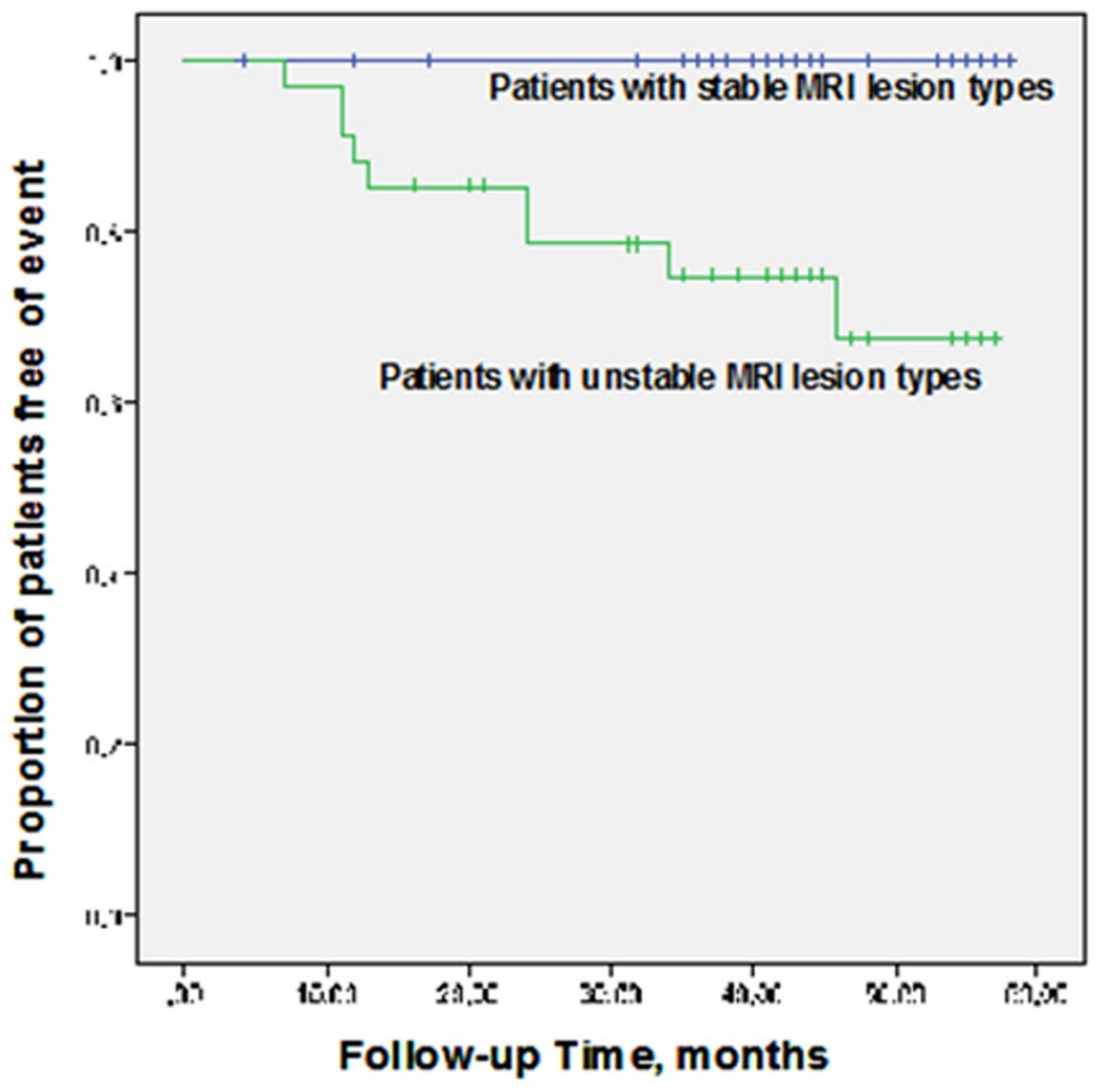
Kaplan–Meier curves. Kaplan–Meier survival estimates of the proportion of patients free of ipsilateral cerebrovascular events for patients presenting with stable MRI lesion types (upper curve) and with unstable MRI lesion types (lower curve). Event-free survival was higher among patients with the MRI-defined stable lesion types (III, VII, and VIII) than in patients with the MRI-defined high-risk lesion types (IV–V and VI) (log rank test *P*<0.0001).

Kaplan–Meier plots for the incidence of ipsilateral cerebrovascular events demonstrated that event-free survival was higher among patients with the MRI-defined stable lesion types (III, VII, and VIII) than in patients with the MRI-defined high-risk lesion types (IV–V and VI) at baseline (58 months event-free probability 100% vs. 67.8%; log rank test *P*<0.0001) (Figure 3).

## Discussion

We found that patients with initially asymptomatic carotid stenosis, in particular patients presenting with the MRI-defined high-risk lesion types IV–V and VI, had significantly higher likelihood of developing associated cerebral events than patients presenting with the stable MRI-defined lesion types. Kaplan–Meier plots for the incidence of ipsilateral cerebrovascular events also demonstrated that event-free survival was higher among patients with the MRI-defined stable lesion types than in those with the MRI-defined high-risk lesion types IV–V or VI at baseline, thus underlining the ability of MRI plaque imaging to detect carotid plaques with a high-risk of future cerebral events.

Our results might be important for clinical purposes because MRI plaque imaging offers a new possibility for noninvasive risk stratification of asymptomatic patients with CS. Identifying asymptomatic patients with CS who are particular at risk of future cerebral events would be of unquestioned importance since these patients could presumably benefit from invasive therapy. Our results suggest that MRI plaque imaging seems to have the potential to identify such asymptomatic but high-risk patients and might help to improve selection criteria for candidates appropriate for intervention procedures.

Our findings are in agreement with the results of a prospective study by Takaya et al. [25], analyzing the relation between asymptomatic MRI plaque features and future stroke: In carotid stenosis they found MRI-deselected high-risk plaque features also to be associated with subsequent cerebral ischemia (n = 154 patients; mean follow-up of 38.2 months). However, they analyzed carotid stenosis with a degree of 50–79%, whereas we investigated stenosis with a degree of 50–99% [29].

Altaf et al. [30] and Lin et al. [31] found MRI-defined vulnerable plaque features such as intraplaque hemorrhage to be related to recurrent cerebral events when analyzing patients with symptomatic CS. Parmar et al. analyzed patients presenting with a symptomatic carotid stenosis and showed that patients with lesion type VI were especially at risk of developing ipsilateral TIA and stroke episodes [32]. These studies analyzed patients presenting with already symptomatic stenosis. However, MRI plaque imaging appears to allow identification of asymptomatic patients who are at risk for future cerebral ischemia and appears to offer the possibility of detecting rupture-prone plaques before they become symptomatic.

MRI-defined vulnerable plaque features such as the presence of intraplaque hemorrhage or a lipid-rich necrotic core were found to be related to recurrent cerebral events when analyzing patients with CS [30], [31], [33], [34], [35]. In a study of Underhill et al., an increasing volume of the lipid-rich necrotic core was related to repeated plaque disruption in previously asymptomatic individuals [36]. However, whereas the above studies evaluated specific predictors of plaque vulnerability such as intraplaque hemorrhage or the volume of the lipid-rich necrotic core, we applied the complete classification of different lesion types introduced by Cai et al. [20] to further broaden the possibility of plaque characterization by considering different plaque subtypes. However, use of the complete modified AHA-classification instead of evaluating distinct established plaque features (e.g., intraplaque hemorrhage) has to be discussed. It has been shown previously that evaluation of intraplaque hemorrhage can be used as a reliable marker for detecting high-risk, rupture-prone plaques [29], [30], [33]. In contrast, the reproducibility for identification of a fibrous cap needs to be improved since intraobserver agreement was only fair for the identification of this plaque feature in the work of Touzé et al. [37]. One could argue, therefore, that evaluation of intraplaque hemorrhage as single plaque feature would allow satisfactory plaque characterization. However, we were especially interested in whether plaque characterization could be further broadened to different lesion types and so wanted to evaluate whether the complete AHA-classification introduced by Cai et al. [20] could be used for risk assessment in patients with carotid stenosis. We wanted to discover whether there is a relation between future cerebral events and distinct lesions types such as lesion type IV–V *and* VI, since to our knowledge there is so far only the work of Takaya et al. [25] analyzing this topic in patients with 50% to79% stenosis, whereas we analyzed patients presenting with 50% to 99% stenosis.

The risk of stroke during follow-up observed in our study (11.7% in 77 patients during a median follow up of 42 months) differs from the stroke rate reported in other studies:

In the study of Goessens et al. [38] only 6 strokes were observed during a mean follow-up of 3.6 years in 221 patients. However, the patients evaluated were younger than ours (∼65 years vs. ∼73 years), which might be an explanation for the differences in the stroke rates observed. Spence and colleagues [39] reported only 2 strokes in 2 years in a population of 269 patients, but the patients in that study received very intensive medical treatment (e.g., maximum tolerated dose of a statin and additionally ezetimibe and/or niacin), whereas the patients in our study received, in addition to an antiplatelet medication, a single statin therapy without additional cholesterol-lowering medication. Marquardt et al. [40] reported a stroke risk of 0.34% per year. In this study a much larger population of patients (n = 1153) was evaluated than in our study, so the differences in the reported stroke rates might also be due to the low number of patients we analyzed.

However, in the study of Nicolaides et al. [41] a stroke risk of approximately 11% was observed in 1117 patients presenting with moderate to severe stenosis. Furthermore, in the study of Halliday and colleagues [42] during a follow-up period of 5 years a stroke risk of 11.8% was reported in 1560 patients presenting with severe carotid stenosis. The stroke rates in both studies were similar to the stroke rate reported in our study.

The studies discussed above all differed regarding study design, patients' demographic factors and the degree of stenosis analyzed, so the comparison of these studies' event rates is difficult.

A limitation of our study is the small patient population and the small number of cerebrovascular events (n = 9; (11.7%) in 77 patients during follow-up). However, since we observed a highly significant relation between MRI-detected vulnerable lesion types and future cerebrovascular events, these results justify larger prospective trials to confirm the ability of MRI to detect endangered atherosclerotic patients while they are still asymptomatic. Several limitations of the MRI technique must be mentioned: MRI plaque imaging is a time-consuming method, and requires the use of expensive carotid surface coils. Moreover, MRI cannot be performed in patients presenting with pacemakers or certain metallic implants. Furthermore, evaluating the MRI images requires a reviewer with advanced experience in MRI plaque imaging. Since in our study four patients (5.2%) had to be excluded because of inadequate MRI image quality, the imaging acquisition process needs to be improved (e.g., by shortening the MRI-scan time to avoid inadequate image quality due to the patient's movements). However, MRI offers a new possibility for non-invasive risk stratification in patients presenting with carotid stenosis and appears to represent a promising technique for the future.

In conclusion, we have shown prospectively that MRI has the potential to identify patients with asymptomatic carotid stenosis who are particularly at risk of developing cerebral ischemia. For the future, MRI-based plaque imaging, as a noninvasive imaging modality, might help to improve risk assessment of carotid artery stenosis and therefore selection criteria of especially endangered patients for invasive therapy.
